# Microscale diamond protection for a ZnO coated fiber optic sensor

**DOI:** 10.1038/s41598-020-76253-5

**Published:** 2020-11-05

**Authors:** Monika Kosowska, Paulina Listewnik, Daria Majchrowicz, Michał Rycewicz, Mikhael Bechelany, Yafit Fleger, Mingzhou Chen, Dror Fixler, Kishan Dholakia, Małgorzata Szczerska

**Affiliations:** 1 Department of Metrology and Optoelectronics, Faculty of Electronics, Telecommunications and Informatics, Gdańsk University of Technology, 11/12 Narutowicza Street, 80-233 Gdańsk, Poland; 2 SUPA, School of Physics and Astronomy, University of St Andrews, North Haugh, St Andrews, KY16 9SS UK; 3 Institut Européen Des Membranes, IEM UMR 5635, University of Montpellier, ENSCM, CNRS, Place Eugène Bataillon, 34095 Montpellier, France; 4 Institute for Nanotechnology and Advanced Materials, Bar-Ilan University, 52900 Ramat-Gan, Israel; 5 Faculty of Engineering, Bar-Ilan University, 52900 Ramat-Gan, Israel

**Keywords:** Optical properties of diamond, Materials for optics, Electrical and electronic engineering

## Abstract

Fiber optic sensors are widely used in environmental, biological and chemical sensing. Due to the demanding environmental conditions in which they can be used, there is a risk of damaging the sensor measurement head placed in the measuring field. Sensors using nanolayers deposited upon the fiber structure are particularly vulnerable to damage. A thin film placed on the surface of the fiber end-face can be prone to mechanical damage or deteriorate due to unwanted chemical reactions with the surrounding agent. In this paper, we investigated a sensor structure formed with a Zinc Oxide (ZnO) coating, deposited by Atomic Layer Deposition (ALD) on the tip of a single-mode fiber. A nanocrystalline diamond sheet (NDS) attached over the ZnO is described. The diamond structure was synthesized in a Microwave Plasma Assisted Chemical Vapor Deposition System. The deposition processes of the nanomaterials, the procedure of attaching NDS to the fiber end-face covered with ZnO, and the results of optical measurements are presented.

## Introduction

Fiber-optic sensors are widely used in environmental, biological and chemical sensing^1–6^. That is due to their well-known advantages that allow for their use in demanding applications – they have the ability to perform real-time and remote measurements, have immunity to electromagnetic interferences and are of compact size, amongst other attributes^7–9^. As new technologies become available, the structures of fiber-optic sensors are growing more elaborate, with integrated thin films, nanoparticles, microstructured fibers, which aim to offer increased sensing abilities. One such group are thin film-based sensors that use nanolayers deposited on the fiber surface^10–12^. These are used to design and create structures that can be applied on the measuring heads of fiber-optic sensors, for example thin (tens of nanometers) dielectric layers made of materials characterized by high refractive index, e.g. ZnO (n = 2.1 at 500 nm)^13,14^.

Using ZnO as a coating for the fiber-optic sensor has many advantages. It allows to broaden the measuring range, improves sensitivity of measured parameter, in comparison to the sensor without a coating. It also enables to perform measurements in a medium, which refractive index is close to this of optical fiber core (n = 1.4). Furthermore, deposition techniques of ZnO are highly developed, especially Atomic Layer Deposition (ALD) method, which ensures uniformity of the coating. However, there are several instances, in which ZnO can adversely affect the surrounding medium, therefore CVD nanocrystalline diamond sheet attached over the ZnO, is required to protect both the sensor head and the measured medium from damage in case of an unwanted reaction.

In addition, fiber-optic sensors are often used in remote places, where other devices cannot be utilized due to their physical dimensions or mechanical properties. By using CVD layer, the sensor can withstand more severe conditions and it can be utilized for longer periods of time, which in turn minimizes involvement of an operator and the cost of maintenance.

Among many extraordinary properties of synthesized diamond, several of those are of particular interest from an optical point of view. It has a very high refractive index (n = 2.4 at a wavelength of 500 nm), and it is transparent in the broad wavelength range. It can also work as a reflective layer when doped with other materials, e.g. boron, nitrogen^15–17^. Diamond is also characterized by its excellent mechanical and chemical properties, as well as biocompatibility. The properties of the diamond can be also tuned by changing the parameters of the deposition process e.g. temperature, time, working gas mixture composition. A set of such outstanding properties resulted in lots of applications of diamond in numerous fields of science, biomedicine and technology^18–21^.

In this work, we investigate an undoped nanocrystalline diamond sheet attached to the ZnO-coated fiber-optic sensor head. The diamond structure was synthesized in the Microwave Plasma Assisted Chemical Vapor Deposition (MW PA CVD) system. The construction of the measurement setup with the focus on the measurement head and the results of the optical measurements are presented. The sensing abilities of the fiber-optic sensor with a ZnO coating deposited by the Atomic Layer Deposition (ALD) method, investigated for temperature change as well as refractive index, are presented elsewhere^10,22^.

### Material characterization—nanocrystalline diamond sheet

**Figure 1:**
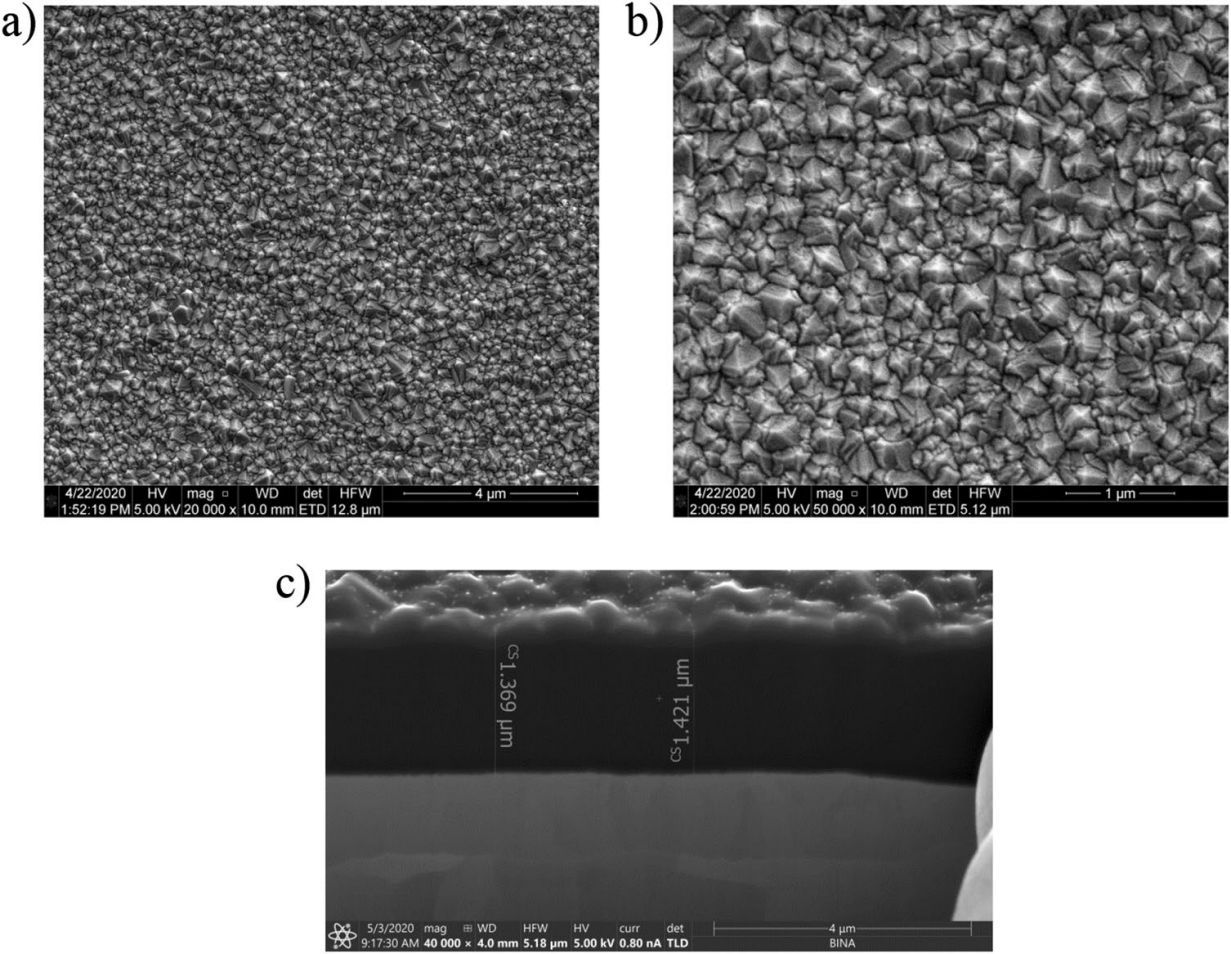
SEM images of nanocrystalline diamond sheet (**a**) magnification 20,000× (**b**) magnification 50,000× (**c**) cross-section magnification 40,000×.

The obtained nanocrystalline diamond sheet surface morphology was investigated with the use of an Environmental Scanning Electron Microscope (E-SEM, Quanta FEG 250, FEI, Hillsboro, Oregon, USA). Figure 1 shows SEM images of the NDS attached to the tantalum substrate for two system magnifications, 20,000× and 50,000×, and for a cross-section magnification of 40,000×.

Figure 1a shows that the diamond had covered the substrate evenly, and over sufficient areas that are large enough for use in the construction of fiber-optic measurement head (with the fiber core dimensions of 8 µm). The obtained structure is uniform and continuous. No damage or other visible abnormalities of the surface were noted during the investigation. Figure 1b proves the crystalline character of the sample, with a grain size smaller than 500 nm. The nanocrystalline diamond sheet was investigated in terms of its thickness, and therefore, cross-section measurements were also taken.

Microscope photographs of the sample were taken with a (C-5060, Olympus, Japan) camera and a (LAB 40 POL, OPTA-TECH, Poland) microscope.

**Figure 2:**
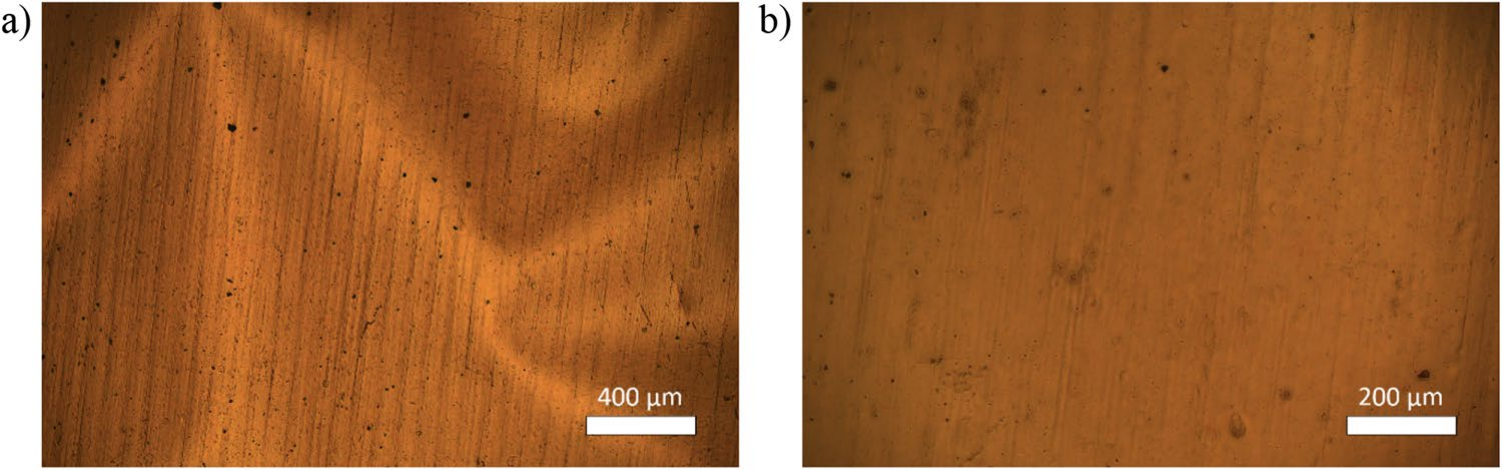
Optical microscope photographs of the diamond sheet (**a**) magnification 50×, (**b**) magnification 100×.

Camera and microscope pictures of a diamond sheet (1 cm × 1 cm in size) are presented in Fig. 2. The rough nanodiamond surface is caused by the delamination process, which facilitates the fabrication of freestanding diamond sheets.

The Raman spectra of the diamond films were measured by using a home-made wavelength modulated Raman system. The details of this system may be found in our previous works^23,24^.

**Figure 3:**
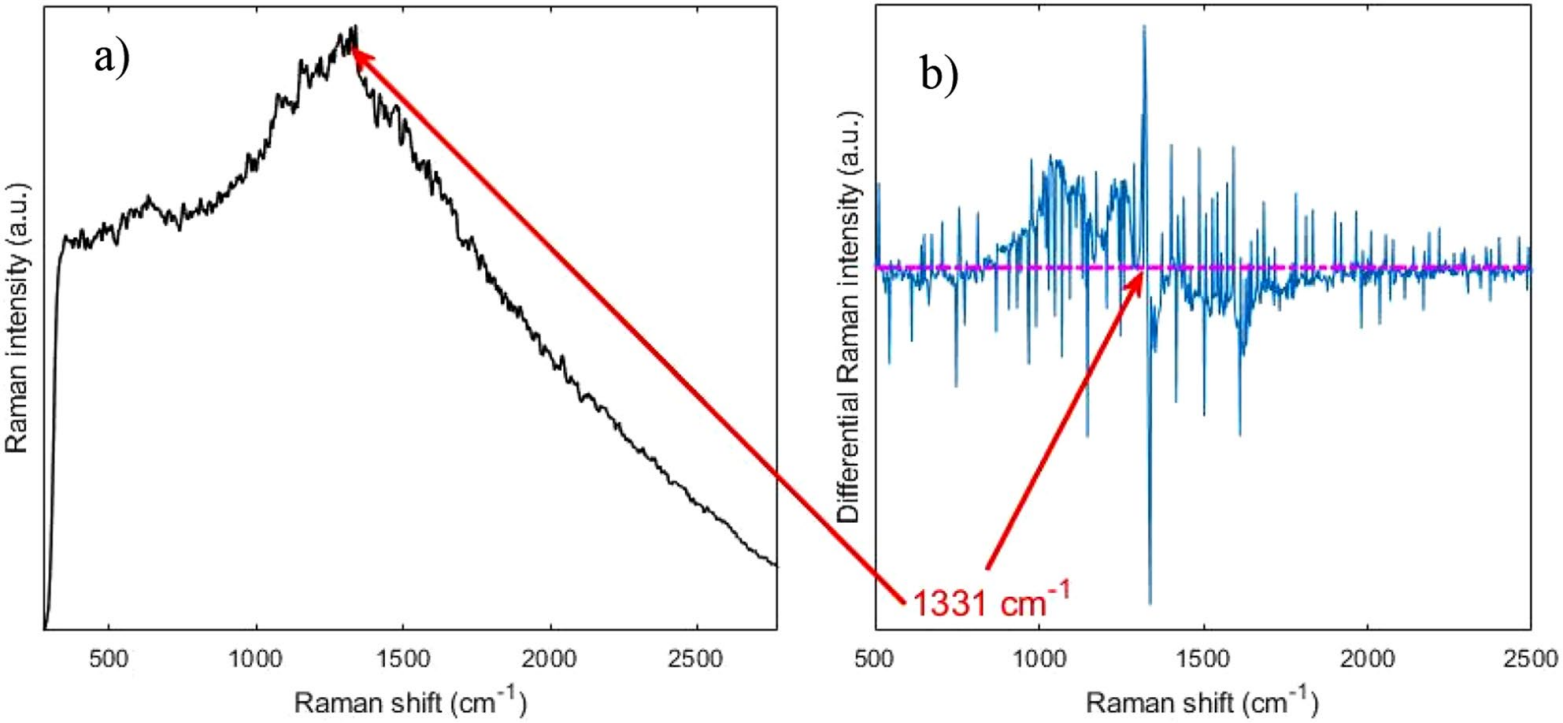
Raman spectra acquired from the nanocrystalline diamond sheet. (**a**) Shows a standard Raman spectrum with a very strong fluorescence background. (**b**) Shows the wavelength modulated Raman spectrum with the 1331 cm^−1^ peak. Arrows point at the zero crossings where the peak is and its possible location in the standard Raman spectrum.

The strong fluorescence background makes it impossible to locate the Raman peaks from a standard Raman spectrum as seen in Fig. 3a. However, our approach of using wavelength modulated Raman system enabled by a tunable narrow linewidth laser can remove the strong fluorescent background efficiently and reveal the underlying Raman features. To achieve this, the wavelength of the laser is tuned in five steps over a range of 1.5 nm from 785 nm. At each step, a Raman spectrum with an integration time of 1 s is recorded. As the fluorescence background does not change over this small wavelength interval, it can be removed with a post-processing algorithm, namely principal component analysis (PCA). This way, the characteristic Raman peak for nanocrystalline diamond sheet at room temperature can be recovered^25,26^, visualized as a zeros-crossing, at 1331 cm^−1^, as shown in Fig. 3b. As this dominant band is assigned to the diamond at room temperature, the investigation proved the successful deposition process resulting in the diamond structure.

## Results

**Figure 4:**
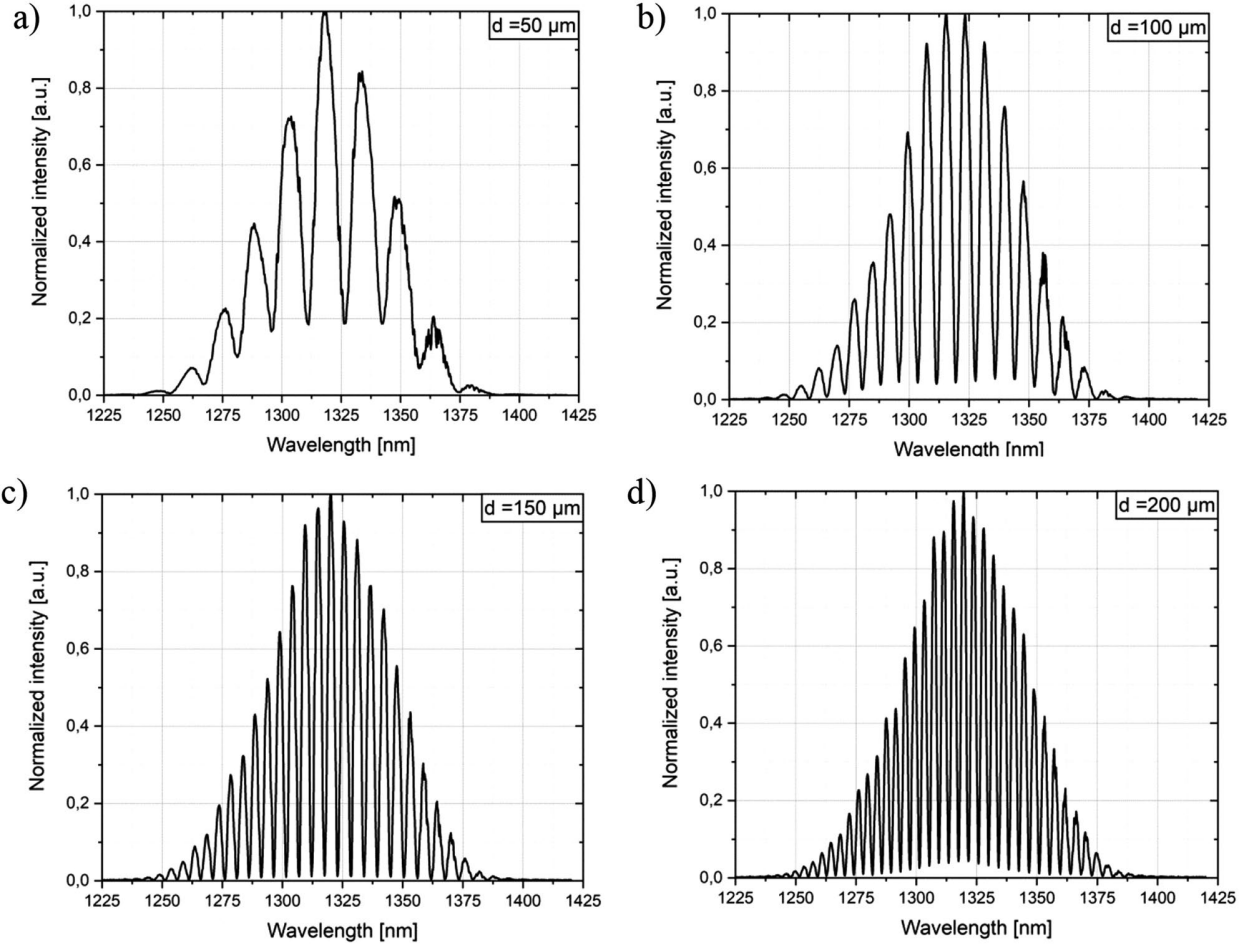
Signal obtained for the measurement head with ZnO layer and nanocrystalline diamond sheet. The Fabry–Perot cavity length was set to: (**a**) 50 µm, (**b**) 100 µm, (**c**) 150 µm, (**d**) 200 µm.

This section contains the experimental measurements, which were performed to evaluate the influence of nanocrystalline diamond sheet attached over ALD ZnO coating. The series of measurements were executed by increasing the distance between the sensor head and the silver mirror by a fixed increment over 500 µm. The measurements were performed using the sensor in two configurations: firstly, with only ZnO coating deposited on the end-face of the fiber, followed by attachment of the NDS and repeating the measurements. The results for both configurations are presented including comparison between them. Figure 4 shows the representative spectra, where the intensity of the obtained signal was normalized. The Fabry–Perot cavity length was set to 50 µm, 100 µm, 150 µm and 200 µm, respectively.

**Figure 5:**
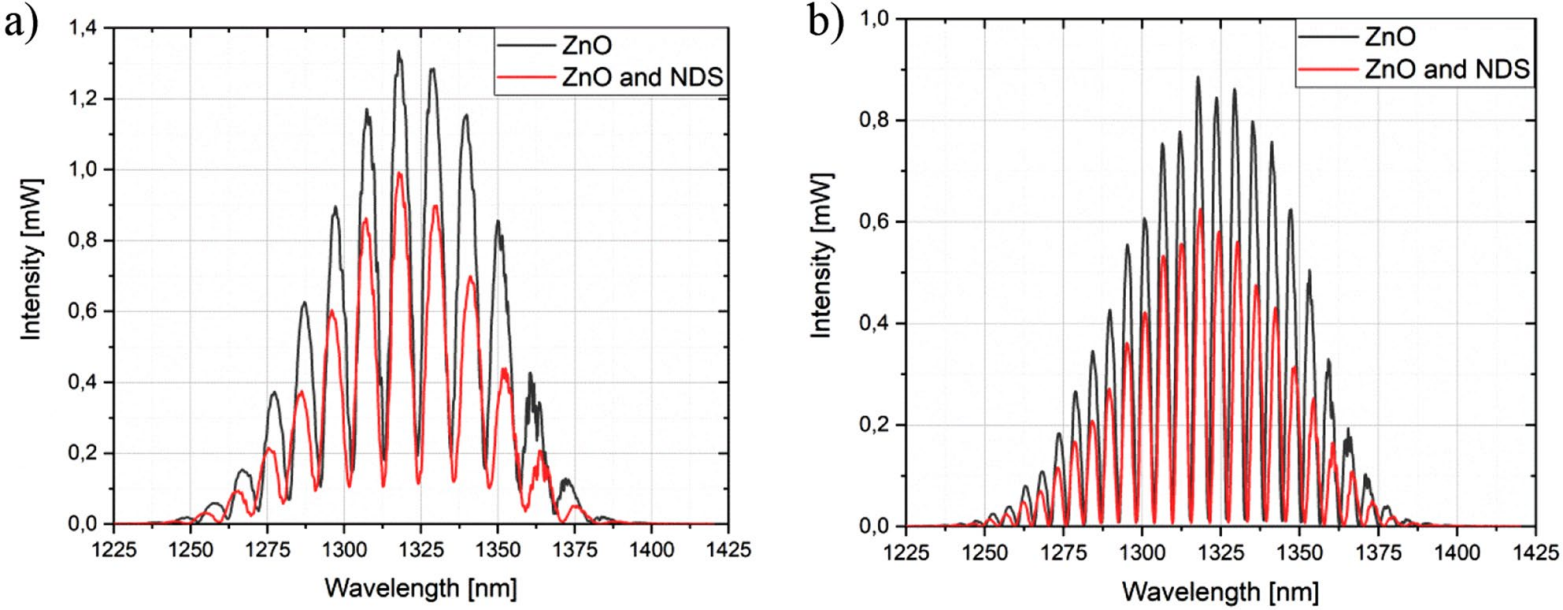
Measurement signals obtained for the measurement head with ZnO coating, and ZnO coating and nanocrystalline diamond sheet. The Fabry–Perot cavity length was set to: (**a**) 70 µm, (**b**) 140 µm.

It can be noted from representative spectra that there is a visible signal modulation while changing the cavity length—the longer the cavity, the greater number of maxima in the investigated wavelength range. This behavior agrees with our previous findings^27^. Figure 5 shows the representative spectra, where the obtained signals for the measurement head with ZnO coating, as well as ZnO coating and nanocrystalline diamond sheet were compared. It can be noted that the intensity of the signal is decreasing while applying the nanocrystalline diamond sheet, which can be observed in Fig. 5. The decrease in the signal intensity does not impact the number of maxima in the spectrum. This demonstrates that NDS provides protection for the fiber end-face covered with a ZnO coating.

The application of the nanodiamond sheets decreases the signal intensity, but the signal visibility is not significantly changed.

The important parameter indicating the contrast of the measurement signal is the visibility value. It can be described with the following formula (1)^28,29^:

1 $$V=\frac{Imax-Imin}{Imax+Imin}$$

where Imax—maximum value of the signal intensity and Imin—minimum value of the signal intensity.

Table 1 presents values of visibility for measurement head with ZnO layer and nanodiamond sheets for different Fabry–Perot cavity lengths.

**Table 1:** Representative values of visibility for different Fabry–Perot cavity lengths.

Cavity length (µm)	Visibility for measurement head with ZnO layer and NDS
50	0.690
80	0.844
110	0.947
140	0.979
200	0.921
300	0.767

## Discussion

To date, only several research groups work on the nanocrystalline diamond sheets, focusing on deposition process, characterization of such structure and methods to transfer the resulting material onto the target surface^30–32^. However, example applications are scarce so far. Bogdanowicz et al. presented the deposition process of a large area NDS, characterization of its properties and demonstration of potential application. This leads to the development of a prototypical, low-temperature diamond-on-graphene transistor^27^. Seshan et al. showed the fabrication process and development of a transfer technique of the obtained diamond sheets with the use of a visco-elastic stamp. The NDS sheets were then used in the fabrication of mechanical resonators^33^.

To the best of author’s knowledge, only our research group reports the application of the NDS in the fiber-optic sensors. The main goal of this study was to investigate the possibility of elaborating a fiber-optic measurement head applying a ZnO coating and the nanocrystalline diamond sheet attached over it as a protection. We showed that such integration can be performed in a simple way, assuring the correct operation of the interferometric sensor. Addition of the NDS provides a protective cover while maintaining the sensing abilities and metrological parameters. Such configuration can extend the lifespan of the sensor and prevent the coating underneath from degradation in challenging environments.

Further investigation regarding the usage of NDS for fiber-optic measurement heads in chemically aggressive media is planned. The comparison of measurement head (with and without NDS) metrological parameters after such experiments, as well as specifying the lifespan difference between them, would be the next step to directly show the benefits of NDS protection.

## Conclusions

In this paper, the fiber-optic measurement head comprising of a single-mode optical fiber, ZnO coating deposited on its end-face, and with an attached nanocrystalline diamond sheet was performed. The results of the optical measurements were described. The study proves that a NDS can be applied as a protection of the fiber-optic end-face. The attachment of the NDS did not influence the sensing abilities of the fiber sensor. This provided protection for the measurement head, which can be exposed to demanding environmental conditions. Furthermore, such protection does not influence meteorological parameters of the fiber-optic sensors because even with the NDS it was able to obtain the visibility of measured signal at the value of 0.979.

On the other hand, maximum visibility can be achieved with a small cavity length at 140 µm, which guarantees the possibility of using very small samples, which is extremely important for example during biological measurement and with the use of biological samples.

## Methods

### Chemical vapor deposition

Nanocrystalline diamond sheets were synthesized in a Microwave Plasma Enhanced Chemical Vapor Deposition (MW PECVD) reactor with a frequency of 2.45 GHz (SEKI Technotron AX5400S, Japan) on mirror-polished tantalum substrates (Sigma-Aldrich Chemie, thickness 0.025 mm, 99.9% metal base) and attached to the sensor head by employing the Van der Waals force. Prior to growth, the substrates were ultrasonically abraded for 30 min in a water suspension consisting of nanodiamond particles of 4–7 nm in diameter, followed by ultrasonic cleaning in acetone and 2-propanol. The temperature of the graphite stage, on which the substrates rested, was kept at 500 °C with a deposition time of 3 h. The microwave power, gas flow rate, and CH_4_:H_2_ molar ratio were 1.1 kW, 300 sccm, and 1%, respectively. The chemical vapor deposition process pressure was maintained at 50 Torr. The detailed description of the deposition process may be found elsewhere^26,27^.

### Fiber-optic interferometer

**Figure 6:**
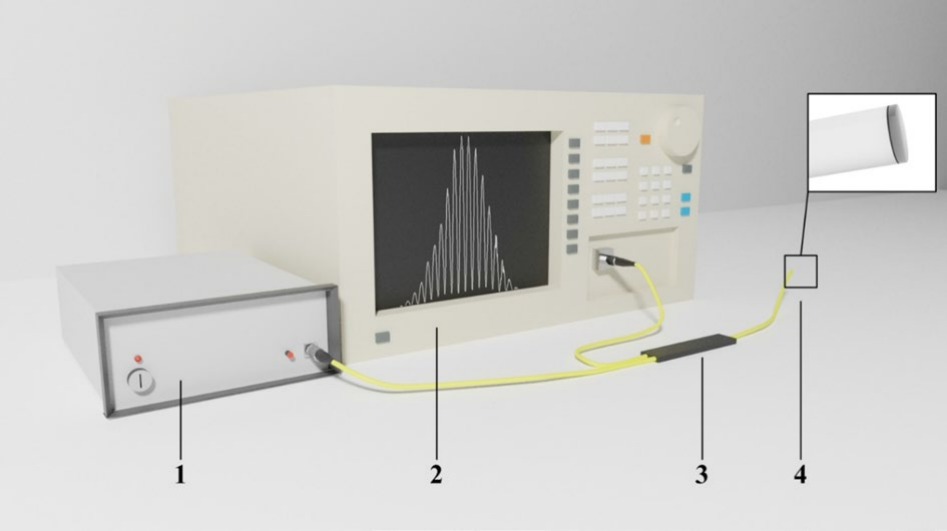
Schematic of the experimental setup, where: 1—light source, 2—Optical Spectrum Analyzer, 3—optical coupler, 4—measurement head.

To verify the sensing parameters of each layer, they were examined in an experimental setup, presented in Fig. 6.

The configuration of the setup was that of Fabry–Perot operated in a reflective mode. A light source with a wavelength of 1310 ± 10 nm—superluminescent diode (SLD-1310-18-W, FiberLabs Inc., Japan) was chosen to perform experimental measurements. The light propagated through the optical coupler and the end-face of the measuring head, and it was reflected of the silver mirror, which was placed directly under the sensor head. The signal reflected from fiber end-face (in configuration ZnO coating and a diamond sheet) and the mirror superpose, resulting in an interference, which can be observed on an Optical Spectrum Analyzer (OSA, AndoAQ6319, Japan). Obtained interferograms can be then analyzed.

### The attachment of the nanocrystalline diamond sheet

**Figure 7:**
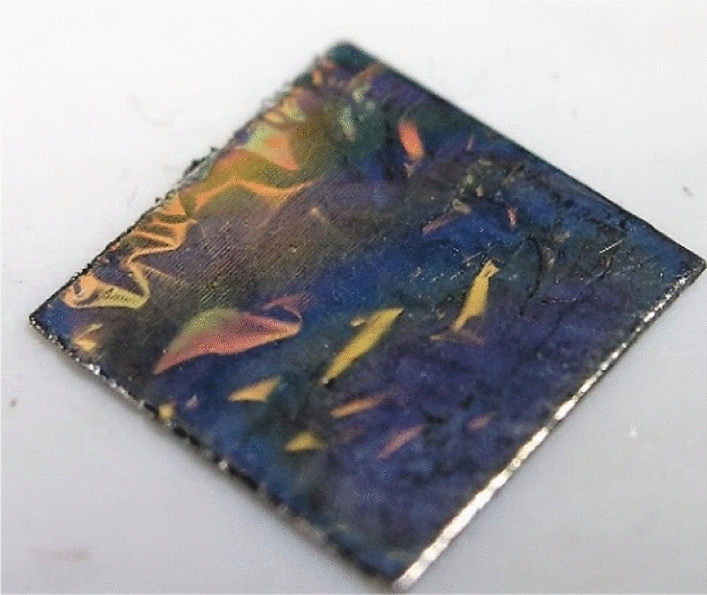
The nanodiamonds structure deposition on the tantalum substrate.

To assess the impact of the nanocrystalline diamond sheet on the obtained signal, NDS was attached over fiber end-face with deposited ZnO. Figure 7 shows the structure of the nanodiamond deposited on the tantalum substrate.

The procedure of the application of the NDS starts with its delamination from the tantalum substrate. The deposition process of the diamond structure was designed in such a way, that the resulting NDS shows low adhesion to the substrate, to ease the process of its release. A scalpel was used to induce stress in the structure in order to achieve a free-standing diamond sheet. Due to this, a part of the sheet detached from the tantalum in the form of an irregular flake which can be transferred onto the desired surface. The NDS was placed on a table of a micromechanical setup, where the fiber-optic measurement head was mounted. The diamond structure was then positioned centrally beneath the fiber. To attach the diamond structure to the measurement head, the van der Waals force was employed by slowly decreasing the distance between the structures, using micrometer screw. The bond was then strengthened by a slight press against the NDS.
